# What Single-Topic Summaries Miss in Hospital Reviews—Aspect-Level Evaluative Structure Using Generative Pretrained Transformer–Based Sentiment Analysis: Content Analysis

**DOI:** 10.2196/92325

**Published:** 2026-09-14

**Authors:** Jung-Tang Hsueh, Sheng-Hsun Hsu, Shwu-Fen Chiu

**Affiliations:** 1 National Kaohsiung Normal University Kaohsiung Taiwan; 2 Department of Business Administration Chung Hua University Hsinchu Taiwan; 3 Center for Continuing Education National Tsing Hua University Hsinchu Taiwan; 4 College of Management Chung Hua University Hsinchu Taiwan; 5 President's Office Dachien General Hospital Miaoli Taiwan

**Keywords:** sentiment analysis, natural language processing, patient satisfaction, quality of health care, hospitals, machine learning, text mining, Taiwan

## Abstract

**Background:**

Health care service quality is inherently multidimensional; yet, the dominant practice in applied text analysis assigns each patient review to a single topic via Latent Dirichlet Allocation (LDA). This simplification may systematically compress evaluative information when patients discuss multiple service dimensions with varying sentiments within the same review.

**Objective:**

This study compared the dominant-topic operationalization commonly used in applied LDA research with Generative Pretrained Transformer (GPT)–based aspect-based sentiment analysis (ABSA) to examine (1) the extent to which patient reviews contain multiple service-quality aspects and how single-topic summaries represent or obscure this structure, (2) the prevalence and patterning of mixed-sentiment reviews, and (3) whether positive and negative reviews differ in aspect comention profiles, before and after adjusting for marginal aspect prevalence.

**Methods:**

We analyzed 5467 Google Reviews posted in 2024 from all 24 medical centers in Taiwan. LDA (K=7 topics) and GPT-based ABSA with structured prompts were applied to the same corpus, with the 7 ABSA categories aligned to the LDA topic labels for a controlled but information-asymmetric comparison. Two independent annotators achieved interrater reliability of Cohen κ=0.82; against the consensus gold standard, GPT-4o achieved an accuracy of 0.89, a weighted *F*_1_ of 0.89, and a Cohen κ of 0.78. Mixed-sentiment reviews were identified as those containing both positive and negative aspect evaluations. Rating-stratified network analysis compared aspect comention patterns between positive and negative reviews using Jaccard similarity, with pointwise mutual information as a prevalence-adjusted sensitivity analysis.

**Results:**

Aspect-bearing reviews discussed an average of 2.05 distinct aspects (95% bootstrap CI 2.02-2.08), yielding an illustrative 51.2% representational compression estimate under dominant-topic assignment (95% bootstrap CI 50.6%-51.9%). A soft-assignment LDA baseline reduced count-level compression to 1.7%, but semantic alignment with ABSA aspects remained limited (mean set Jaccard=0.33), and topic assignments carry no aspect-level sentiment polarity. Among multiaspect reviews, 11.0% exhibited cross-aspect mixed sentiment, with Technical-Functional Divergence—praising technical quality while criticizing functional quality—appearing in 61.6% of these cases. Clinical dimensions were more frequently comentioned in positive reviews and operational dimensions in negative reviews; however, pointwise mutual information analysis indicated that these differences were substantially confounded with marginal aspect prevalence rather than reflecting differential co-occurrence tendencies.

**Conclusions:**

In this corpus, dominant-topic assignment compressed multiaspect patient feedback; soft-assignment LDA recovered topic counts but did not restore semantic alignment or aspect-level sentiment polarity. A nontrivial subset of reviews exhibited cross-aspect mixed sentiment, most commonly praising clinical competence while criticizing functional service dimensions, and positive and negative reviews discussed different constellations of quality dimensions—differences that primarily reflect which aspects patients discuss rather than prevalence-independent associations. Aspect-level analysis that preserves both multidimensional structure and sentiment polarity may help organize patient feedback at a more diagnostically specific level than single-topic summaries.

## Introduction

Understanding patient experiences is central to health care quality improvement and organizational learning. As health systems increasingly turn to digital data sources for performance monitoring, online reviews on platforms such as Google Maps have become an important complement to structured patient surveys. These unsolicited narratives provide granular insights into patients’ lived experiences, allowing them to articulate strengths and shortcomings of care in their own words [1,2]. However, the unstructured nature of such feedback poses substantial challenges for systematic analysis and limits its effective use in decision support for quality improvement.

Health care service quality is inherently multidimensional. The SERVQUAL (Service Quality) framework identifies key dimensions of perceived service performance [3] and has been widely adapted in health care contexts [4]. Donabedian’s structure-process-outcome model further distinguishes organizational context, care delivery processes, and patient outcomes [5]. A systematic review of 37 validated patient satisfaction instruments confirms that satisfaction is consistently operationalized as a multidimensional construct, with convergence around patient-provider interactions, physical environment, and management processes [6]. This multidimensionality implies that quality-monitoring applications may benefit from preserving multiple co-occurring quality dimensions within individual narratives rather than reducing evaluations to a single dominant theme. Moreover, the evaluative space model posits that positive and negative appraisals can be coactivated rather than canceling into neutrality [7], suggesting that patients may simultaneously praise and criticize different dimensions of the same encounter—a pattern that has recently begun to be operationalized as aspect-level sentiment ambivalence [8].

Latent Dirichlet Allocation (LDA) [9] has become a widely used method for extracting thematic structures from patient feedback, particularly because its unsupervised nature enables large-scale analysis without manual annotation. Prior applications include analyses of health-related blogs [10], clinical content on social media [11], and longitudinal assessments of hospital performance [12]. A systematic review of topic modeling in user reviews confirms that LDA remains the dominant method across application domains [13]. Although LDA is formally a mixed-membership model that represents each document as a distribution over all topics, the dominant practice in applied research is to assign each document to its single highest-probability topic. This dominant-topic simplification is the focus of our comparison. Several extensions partially relax this convention: sentence-level topic models assign topics per sentence rather than per document, allowing multiple themes within a single text [14]; and joint sentiment-topic models jointly estimate topics and their associated sentiment polarities within a single generative framework [15]. Nevertheless, dominant-topic assignment remains the common practice, and the representational consequences of this convention have received limited scrutiny.

Under dominant-topic assignment, each review is characterized by a single primary theme, implicitly assuming that one topic adequately represents the patient’s narrative. For health care quality monitoring, this assumption is problematic. Consider a patient stating: “The surgeon was professional and thorough, but I waited three hours in the emergency department, and the registration staff were unhelpful.” This review simultaneously addresses clinical professionalism (positive), emergency waiting time (negative), and administrative attitude (negative). When such multiaspect, mixed-sentiment evaluations are reduced to a single topic, clinically and managerially relevant evaluative structure may be obscured. Soft-assignment alternatives—such as retaining all topics above a probability threshold—can partially address the count-level gap, but they do not resolve the absence of aspect-level sentiment polarity. What remains underexamined in health care contexts is how single-topic summaries differ from aspect-level representations in preserving aspect diversity, sentiment polarity, and comentioned service dimensions.

Joint sentiment-topic models partially address this sentiment gap [15], but they typically operate at document-topic levels rather than extracting explicit aspect-opinion pairs. Aspect-based sentiment analysis (ABSA) offers an alternative paradigm by treating each mentioned aspect as an independent unit of analysis, simultaneously identifying the aspect category, the opinion expression, and its sentiment polarity [16,17]. ABSA research has evolved from supervised deep learning approaches to fine-grained multielement extraction, with recent work extending to quadruple analysis that jointly extracts target, aspect, opinion, and sentiment [18]. In health care, ABSA has demonstrated potential for decision support applications, such as ranking hospitals by aspect-level sentiment [19].

However, conventional ABSA approaches typically require extensively annotated training data, limiting scalability and cross-domain adoption. Recent advances in large language models (LLMs), particularly the Generative Pretrained Transformer (GPT) family, offer new opportunities for guided or few-shot aspect extraction through natural language instructions, category definitions, and worked examples [20]. In hospitality management, zero-shot text classification using pretrained natural language inference–based transformer models has been applied to ABSA, reducing reliance on domain-specific labeled training data while preserving sentence-level aspect-sentiment pairing [21]. In health care, LLM-based ABSA has been used to analyze patient reviews, identifying key drivers of satisfaction at the aspect level [22-24]. However, LLM-based approaches raise concerns about prompt sensitivity, model version instability, output hallucination, and reproducibility [25,26], underscoring the need for structured validation when applying these methods to health care decision support.

To examine these representational consequences, we compare dominant-topic LDA with GPT-based ABSA on the same corpus of hospital reviews. This comparison is information-asymmetric by design: GPT-based ABSA receives a predefined 7-category taxonomy with definitions and worked examples, whereas LDA is run unsupervised. Holding the label set constant ensures that observed differences reflect representational capacity (dominant-topic vs multiaspect assignment) rather than taxonomic differences, but it does not hold input information constant. The comparison therefore evaluates what becomes visible when reviews are represented as aspect-sentiment configurations rather than single-topic summaries, not whether GPT-based ABSA is inherently superior to LDA for all analytic purposes.

Despite growing recognition that analytic granularity shapes the insights derived from patient-generated text, 3 critical gaps remain. First, limited research has examined how dominant-topic representations affect the preservation of aspect diversity and aspect-level evaluative structure in health care reviews. Second, dominant-topic assignment does not directly represent mixed sentiment, as it lacks aspect-level polarity. Consequently, the prevalence of evaluative tensions—where patients simultaneously praise and criticize different aspects of care—and their distribution across service quality dimensions remain insufficiently examined. Third, less is known about whether positive and negative reviews differ in their observed aspect co-mention profiles, or whether such patterns primarily reflect shifts in marginal aspect prevalence. Addressing these gaps can advance text-analytic approaches that meaningfully support health care quality improvement. Specifically, this study addresses 3 research questions:

- Research question 1: To what extent do patient reviews contain multiple service-quality aspects, and how are these aspect-level structures represented or obscured by single-topic summaries?

Representational compression is not the only concern. Dominant-topic representations also omit evaluative polarity: they identify *what* patients discuss but not *how* patients evaluate each dimension. Consequently, they do not directly represent mixed evaluations. Such trade-offs are central to service-quality theory because they reveal when technical quality (*what* is delivered) and functional quality (*how* it is delivered) can diverge within the same encounter [27]. Beyond cross-aspect trade-offs, patients may also evaluate different service providers within the same quality dimension differently—praising one staff member while criticizing another. Capturing these tensions requires pairing aspect identification with sentiment polarity, motivating our second research question:

- Research question 2: How prevalent are mixed-sentiment reviews, and what quality tensions do they indicate—both cross-aspect trade-offs and within-aspect ambivalence?

Beyond individual review characteristics, an open question is whether the aspect comention profiles of patient evaluations differ between satisfied and dissatisfied patients, and whether such differences reflect genuine co-occurrence tendencies or shifts in marginal aspect prevalence, motivating our third research question:

- Research question 3: Do positive and negative reviews exhibit different aspect comention profiles, and to what extent do these observed patterns persist after adjusting for marginal aspect prevalence?

## Methods

This section first describes the study design and data collection and then details the 2 analytic methods—LDA topic modeling and GPT-based ABSA—applied to the same corpus of 5467 patient reviews. It concludes with the comparison framework used to examine aspect diversity and single-topic representation, characterize mixed-sentiment reviews, and analyze aspect co-occurrence patterns across rating groups.

### Study Design

This study used a comparative analytical design to assess the information captured by LDA versus GPT-based ABSA when analyzing health care patient reviews. Both methods were applied to the same 5467 reviews from 24 medical centers in Taiwan. This design held the corpus and 7-category label set constant, but it was not information-symmetric: GPT-ABSA used predefined category definitions and worked examples, whereas LDA was estimated in an unsupervised manner.

The analysis consisted of 3 phases. Phase 1 applied LDA with K=7 topics, assigning each review to its dominant topic. Phase 2 applied ABSA using GPT with structured prompts to map reviews to 7 aspect categories derived from the LDA topic structure. Phase 3 conducted systematic comparisons analyzing aspect diversity, mixed-sentiment detection, and aspect co-occurrence network analysis stratified by rating group.

### Data Collection

Patient reviews were retrieved from Google Maps because the platform provides (1) consistent coverage across health care institutions, (2) verified linkage between reviews and specific hospital locations, and (3) a standardized 1-5 star rating structure. Because Google Maps displays review time stamps in relative terms (eg, “3 months ago”) rather than absolute posting dates, we recorded the displayed relative time and the time stamp of each data collection session and then converted the relative time to an approximate calendar date to retain reviews posted within the study window (January 1 to December 31, 2024). Data were retrieved in January 2025 using Python-based automated scripts (Selenium). This relative-to-absolute conversion introduces potential boundary misclassification near January 1 and December 31, 2024. Because Google Maps’ relative time stamps have coarser granularity at longer intervals (eg, “1 year ago” rather than “12 months ago”), reviews near the boundaries may be misassigned by up to several weeks. This imprecision may have included a small number of late-2023 or early-2025 reviews or excluded borderline 2024 reviews, but it is unlikely to systematically bias the thematic or sentiment patterns analyzed in this study.

We focused on medical centers, the highest tier in Taiwan’s hospital accreditation system. In Taiwan, hospitals are accredited by the Ministry of Health and Welfare and categorized into medical centers, regional hospitals, and district hospitals. Medical centers serve as tertiary referral and teaching hospitals with comprehensive specialty services and advanced emergency and critical care capacity. We included all 24 Ministry of Health and Welfare–accredited medical centers. Each record included hospital identifier, overall star rating (1-5), and the verbatim review text.

From 8547 collected reviews, we sequentially excluded 2833 reviews with missing or empty text (including star-only ratings without accompanying text) and 247 reviews that yielded no valid tokens after segmentation and filtering (eg, symbols-only or stopwords-only), resulting in a final analytic sample of 5467 reviews (mean length 106.9 characters, SD 169.5). We did not apply additional short-text filtering thresholds (eg, minimum character length or minimum token count) for 2 reasons: first, short reviews represent authentic patient feedback that hospitals encounter in practice; second, retaining them allows fair evaluation of how each method handles sparse content—specifically, dominant-topic assignment versus ABSA’s ability to recognize content-free reviews.

The star rating distribution in the final sample exhibited a pronounced U-shaped pattern, with a slight majority of negative reviews (1-2 stars: 51.0%) compared with positive reviews (4-5 stars: 45.2%).

### LDA Document-Level Analysis: Preprocessing

Chinese text preprocessing used Jieba for word segmentation with a custom medical dictionary containing health care–specific terms across 5 categories: clinical departments (eg, Obstetrics and Gynecology, Otolaryngology), medical staff roles (eg, registered nurse, front-desk staff), service processes (eg, registration, billing), hospital facilities (eg, intensive care unit, waiting room), and medical procedures (eg, CT scan, endoscopy). Standard segmenters often break multicharacter medical terms incorrectly; our dictionary ensured that these compound terms were treated as single tokens. For code-mixed English or Chinese text, Jieba’s default behavior treats contiguous American Standard Code for Information Interchange sequences as single tokens during segmentation, so multicharacter English abbreviations (eg, “MRI” and “CT”) were not split. After segmentation, English and Chinese tokens were subjected to the same filtering rules: a custom stopword list removed high-frequency function words (eg, de, le) and domain-general terms (eg, hospital, feel), and single-character tokens were removed regardless of script.

### Model Specification and Topic Assignment

The LDA model was implemented using Gensim 4.3.0 with K=7 topics and was trained on the full analytic corpus of 5467 reviews, including the 749 reviews that the ABSA pipeline later flagged as containing no service-quality content. Retaining these reviews in the LDA training set was a deliberate choice: it simulates how dominant-topic assignment operates on real-world corpora where off-topic or content-sparse documents are not prefiltered; moreover, LDA must assign every document a topic distribution regardless of content. We acknowledge that including these reviews may slightly reduce topic coherence. The Gensim dictionary was filtered to retain only tokens appearing in at least 5 documents and no more than 50% of the corpus. We evaluated candidate topic numbers (K=2-15) using the C_v coherence metric (Figure S1 in Multimedia Appendix 1). Coherence was highest at K=2 (C_v=0.518), reflecting the tendency of low-K solutions to concentrate high-frequency co-occurring terms into broad clusters that collapse multiple service quality dimensions into undifferentiated positive versus negative groupings. For K≥5, coherence values entered a relatively stable range (C_v=0.426-0.491), indicating diminishing returns from additional topics. A narrow local increase at K=12 (C_v=0.491) was not sustained at adjacent values (K=11: 0.426; K=13: 0.457), suggesting a local coherence fluctuation rather than a more parsimonious or substantively preferable topic structure. Within this range, K=7 represented a local maximum (C_v=0.486) and yielded 7 semantically coherent topics corresponding to distinct health care service quality dimensions (Table 1). Parameters included symmetric alpha, automatic eta, 100 iterations, 10 passes, and random_state=42 for reproducibility.

**Table 1:** Latent Dirichlet Allocation topics (K=7).

Topic	Label	Top 5 keywords	Reviews, %	Average rating
T1	Service Attitude Issues	Attitude, hospital, patient, staff, and know	20.3	1.67
T2	Administrative Processes	Time, consultation, registration, doctor, and appointment	13.9	1.69
T3	Professional Quality	Physician, examination, patience, surgery, and thank	7.5	4.38
T4	Emergency Care	Doctor, patient, emergency, hospital, and nurse	18.7	1.94
T5	Inpatient Care	Thank, staff, professional, ward, and nurse	25.5	4.69
T6	Surgical and Specialty Care	Surgery, treatment, medical, hospital, and physician	5.3	2.93
T7	Facility and Environment	Hospital, center, payment, convenient, and equipment	8.7	3.10

Topic labels were assigned based on dominant keyword themes and validated through examination of high-probability representative reviews. Most topics exhibited clear semantic coherence: T1 centered on attitude-related complaints, T2 on registration and scheduling processes, T3 on physician expertise and patient gratitude, T4 on emergency department experiences, T5 on inpatient ward care and nursing, and T6 on surgical procedures. T7 presented more heterogeneous keywords; however, representative review analysis revealed consistent themes around hospital facilities and environmental factors (eg, parking availability, hospital layout, and equipment), justifying the “Facility and Environment” label. Each review in the LDA analytic sample was assigned to its dominant topic based on maximum posterior probability, following standard dominant-topic assignment.

### Aspect-Based Sentiment Analysis

#### ABSA Design

The 7 ABSA aspect categories were deliberately derived from the LDA topic structure as a consequential design decision. K=7 was selected from a coherence plateau (K≥5) as a local maximum that yielded semantically interpretable health care quality dimensions (see Model Specification and Topic Assignment section); the 7 ABSA categories were then defined to match these topics. This circularity is intentional: by holding the evaluative taxonomy constant, any observed differences between LDA and ABSA reflect representational capacity (dominant-topic vs multiaspect assignment) rather than taxonomic differences. A consequence of this design is that the downstream comparison partly rests on showing that 2 label sets engineered to be identical nonetheless align only weakly at the review level. The 7 categories are Emergency Care, Professional Quality, Service Attitude, Administrative Processes, Facility and Environment, Surgical and Specialty Care, and Inpatient Care. Post hoc, these categories align with established health care service quality frameworks. They map onto the SERVQUAL dimensions of assurance, empathy, responsiveness, reliability, and tangibles [3]; Donabedian’s structure-process-outcome triad [5]; and the aspect framework consolidated from established health care quality frameworks and applied through LLM-based ABSA to Google Maps urgent care reviews [22], which organized patient evaluations into 5 dimensions: interpersonal factors, technical quality, operational efficiency, finances, and facilities. The convergence between our data-driven categories and these theoretical frameworks suggests that the LDA-derived topics capture substantively meaningful service quality dimensions.

Structured prompts mapped each review to these 7 aspect categories. All API calls were made on November 30, 2025, using the OpenAI API via Python (Python Software Foundation) with model identifier gpt-4o, which resolved to snapshot gpt-4o-2024-08-06 at the time of execution. Temperature was set to 0 to maximize output determinism. For each identified aspect, the model returned a JSON object containing aspect_category, opinion (verbatim excerpt), sentiment (positive, neutral, and negative), sentiment_score (–1, 0, and +1), and a model-reported confidence score (0.0-1.0). Confidence scores were collected as a diagnostic field rather than as a criterion for primary inclusion; we conducted a sensitivity check by recomputing mixed-sentiment prevalence after filtering review-aspect combinations below selected confidence thresholds. Multiple aspects per review were allowed; the same aspect category could also appear multiple times with different opinion terms (eg, a review praising one nurse’s attentiveness while criticizing another’s rudeness would generate 2 Service Attitude mentions with opposing sentiment). These repeated mentions were retained for mixed-sentiment analysis (research question 2), where within-aspect sentiment variation is substantively meaningful; for aspect diversity counts (research question 1), each aspect category was counted once per review regardless of the number of mentions. For descriptive aspect-level sentiment summaries (Table 2), individual mentions were aggregated to unique review-aspect combinations. When the same review mentioned the same aspect multiple times, the sentiment scores were averaged, and the review-aspect pair was classified as positive, neutral, or negative according to the sign of the mean score. We report positive, neutral, and negative proportions per aspect category as the primary summary, retaining the arithmetic mean of sentiment scores only as a secondary index.

**Table 2:** Aspect distribution and sentiment composition^a^.

Aspect category	Review-aspect combinations	Values, %	Positive, %	Neutral, %	Negative, %	Mean sentiment
Service Attitude	2884	29.8	42.4	2.9	54.7	–0.12
Professional Quality	2495	25.8	57.6	3.8	38.6	+0.20
Administrative Processes	1371	14.2	11.6	4.1	84.3	–0.72
Facility and Environment	877	9.1	30.7	3.8	65.6	–0.34
Inpatient Care	842	8.7	54.0	7.1	38.8	+0.16
Emergency Care	626	6.5	21.6	20.6	57.8	–0.36
Surgical and Specialty Care	578	6.0	60.9	12.3	26.8	+0.33
Total	9673	100.0	41.7	5.5	52.9	–0.10

^a^Each row counts unique review-aspect combinations; when the same aspect category appeared multiple times within a single review (eg, praising one nurse while criticizing another), sentiment scores were averaged to assign a single positive, neutral, or negative label to that review-aspect pair (15,752 individual mentions aggregated to 9673 review-aspect combinations). Positive, neutral, and negative percentages are calculated within each aspect category. Mean sentiment is retained as a secondary summary index; the proportional breakdown is the primary representation because averaging ordinal scores (–1, 0, and +1) can obscure polarization. Mean sentiment is computed over the averaged continuous scores for each review-aspect pair, whereas the positive, neutral, and negative proportions reflect the sign of those averaged scores. The 2 columns are therefore not arithmetically interchangeable, and the mean should not be reconstructed from the proportions.

#### Handling Nuance and Sarcasm

LLMs may support contextual interpretation of figurative language, including sarcasm, which is common in negative reviews. The prompt explicitly instructed the model to treat positive wording, followed by complaints or criticism as potentially sarcastic or ironic. For example, if a patient writes “Thanks for making me wait 4 hours!,” the model was directed to classify these as negative Administrative Processes rather than positive expressions of gratitude. This type of contextual nuance is difficult to capture with traditional bag-of-words approaches.

#### Prompt Engineering and Quality Validation

The API was configured to enforce structured JSON output. If a response failed JSON parsing, the pipeline automatically retried up to 3 times before flagging the review for manual inspection. Postretrieval validation checked (1) conformance to the JSON schema, (2) use of valid aspect categories, and (3) sentiment scores within the allowed set (–1, 0, and +1). Reviews failing validation were excluded from analysis. In practice, all 5467 API calls returned valid JSON. The complete prompt design is provided in Multimedia Appendix 1. The prompt required GPT-4o to return verbatim opinion spans alongside aspect and sentiment labels. Validation evaluated aspect detection accuracy and sentiment classification accuracy against the gold standard, but span extraction accuracy was not independently validated. The opinion spans serve as interpretive aids for qualitative illustration rather than as validated analytic outputs.

Quality validation used 201 reviews drawn by stratified random sampling by star rating, independently coded by 2 trained annotators using the same 7 aspect categories and the 4-level coding scheme (absent, negative, neutral, and positive) for each review-aspect pair. Interrater reliability between the 2 human coders achieved Cohen κ=0.82. Disagreements were resolved through discussion between the 2 coders until consensus was reached; no third adjudicator was used. The consensus outcome was designated as the gold standard. Note that κ=0.82 is a chance-corrected agreement measure; the corresponding raw agreement rate was higher. Using the consensus outcome as the gold standard, GPT-4o validation performance is reported in the Results section.

### Analytical Comparison Framework

#### Quantification of Representational Compression

Aspect diversity was calculated as distinct aspect categories per review. Under dominant-topic assignment, each review receives exactly 1 topic label; under soft-assignment baselines, each review could receive multiple topic labels based on posterior probability thresholds or top-k assignment. Compression was operationalized descriptively as (Mean_ABSA – Mean_LDA)/Mean_ABSA, where Mean_ABSA denotes the mean number of GPT-derived aspects per review and Mean_LDA denotes the mean number of LDA topics retained per review under the assignment method being evaluated. The primary compression calculation was restricted to the 4718 reviews with at least 1 identifiable service quality aspect; a denominator sensitivity check repeated the calculation after adding the 749 zero-aspect reviews. Bootstrap 95% CIs for Mean_ABSA and the compression estimate were computed using 10,000 review-level resamples.

#### Characterization of Mixed-Sentiment Reviews

We operationalized mixed sentiment in 2 ways. Cross-aspect mixed reviews contained at least 1 positively evaluated aspect (sentiment score >0) and at least 1 negatively evaluated aspect (sentiment score <0) within the same review (trade-offs across dimensions). Within-aspect mixed reviews contained both positive and negative opinions within the same aspect category. Neutral aspects (sentiment score=0) did not contribute to either the positive or negative condition in mixed-sentiment classification; they were counted in aspect diversity (research question 1) and included in co-occurrence networks (research question 3) but did not trigger mixed-sentiment flags.

To characterize cross-aspect mixed-sentiment reviews, we compared them with multiaspect reviews lacking mixed sentiment using Mann-Whitney *U* tests on aspect diversity, review length, and star ratings. This comparison excludes single-aspect reviews, ensuring that both groups contain reviews discussing multiple dimensions.

To identify recurring trade-offs, we computed cross-polarity couplings within cross-aspect mixed-sentiment reviews. Each coupling pairs a positively evaluated aspect with a negatively evaluated aspect. With 7 aspect categories, there are 42 possible ordered pairings (7 × 6, excluding same-aspect pairs), ranked by frequency (review count). Because a review could contain multiple positively and negatively evaluated aspects, a single review could contribute to multiple ordered pairings.

For within-aspect mixed sentiment, we reported the mixing rate for each aspect category, calculated as reviews exhibiting within-aspect mixing divided by total reviews mentioning that aspect.

We define Technical-Functional Divergence as a cross-aspect mixed-sentiment review in which at least 1 technical aspect (Professional Quality and Surgical and Specialty Care) is evaluated positively and at least 1 functional aspect (Service Attitude, Administrative Processes, Facility and Environment, Emergency Care, and Inpatient Care) is evaluated negatively within the same review. This classification follows the technical-functional quality distinction [27], where technical quality refers to what is delivered (clinical competence and treatment outcomes) and functional quality refers to how it is delivered (interpersonal interactions, administrative processes, and physical environment).

Because aspect detection recall ranged from 0.67 to 0.90 across aspects (Table 3), some mixed-sentiment reviews may have been classified as pure sentiment if GPT-4o missed the only positive or negative aspect. To estimate the magnitude of this potential undercount, we conducted a recall-gap sensitivity analysis using a Horvitz-Thompson-style correction. For each detected mixed-sentiment review, we estimated its probability of being observed from the per-aspect recall values in Table 3 and then computed the inverse-probability–weighted prevalence estimate. We report both the observed prevalence and the recall-corrected estimate; the denominator for cross-aspect mixed-sentiment prevalence is reported as both a proportion of multiaspect reviews (n=3182) and of all aspect-bearing reviews (n=4718) to avoid ambiguity.

**Table 3:** Per-aspect validation performance (GPT-4o vs gold standard)^a^.

Aspect	Precision	Recall	*F*_1_	Sentiment accuracy	n (sentiment)
Service Attitude	0.96	0.81	0.88	0.94	122
Professional Quality	0.93	0.85	0.89	0.88	98
Administrative Processes	0.91	0.67	0.77	0.95	58
Inpatient Care	0.98	0.89	0.93	0.83	46
Facility and Environment	0.94	0.80	0.86	0.84	43
Surgical and Specialty Care	0.96	0.68	0.79	0.76	25
Emergency Care	0.82	0.90	0.86	0.83	18

^a^Precision, recall, and *F*_1_ refer to binary aspect detection (present vs absent). Sentiment accuracy is the proportion of correct sentiment labels among review-aspect pairs where both GPT-4o and the gold standard detected the aspect. Values are rounded to 2 decimal places.

#### Statistical Analysis

Validation performance was assessed using accuracy, precision, recall, weighted *F*_1_, and Cohen κ against the gold-standard annotations. The validation was decomposed into 2 stages: binary aspect detection (present vs absent) and conditional sentiment agreement (among review-aspect pairs where both GPT-4o and the gold standard detected the aspect). For the primary GPT-4o validation, bootstrap 95% CIs for κ were computed using 2000 resamples of the validation sample. To assess model dependence, we repeated the ABSA extraction on the 201 validation reviews using 2 alternative frontier models from different providers—DeepSeek-V4-Flash nonthinking mode (DeepSeek; accessed through the legacy API identifier deepseek-chat) and Claude Sonnet 4.6 (Anthropic)—applying the identical prompt template and computing the same 4-class, aspect-detection and conditional sentiment agreement metrics against the gold standard. Mean set Jaccard was defined as (1/*n*)Σᵢ|S(LDA,*i*) ∩ S(ABSA,*i*)| / |S(LDA,*i*) ∪ S(ABSA,*i*)|, where S(LDA,*i*) denotes review *i*’s set of soft-LDA topic labels and S(ABSA,*i*) denotes its set of GPT-derived aspect labels, using the topic-to-aspect label mapping in Table 1. For group comparisons of mixed-sentiment review characteristics (research question 2), Mann-Whitney *U* tests were used with rank-biserial correlation (*r*) as effect size. For the co-occurrence network analysis (research question 3), bootstrap 95% CIs for Jaccard differences were computed using 2000 review-level resamples within each rating group, and statistical significance was assessed using 5000 label-permutation tests (permuting the rating-group assignment of reviews) with Benjamini-Hochberg false discovery rate correction across all 21 aspect pairs.

#### Aspect Co-Occurrence Networks by Rating

To examine whether high- and low-rated reviews organize aspect mentions differently, we constructed rating-stratified aspect co-occurrence networks. Reviews rated 4-5 stars were defined as positive reviews and reviews rated 1-2 stars as negative reviews; 3-star reviews were treated as neutral and excluded.

Within each network, aspects were connected using the Jaccard similarity coefficient, J(A,B)=|A∩B|/|A∪B|, where A and B denote the sets of reviews that mention each aspect. With 7 aspect categories, there are 21 possible aspect pairs. Networks were compared by computing Δ=Jaccard(Positive) – Jaccard(Negative) for each pair, identifying core pairings (strong in both groups) and large raw Jaccard differences between rating groups. Robustness was assessed by repeating the analysis using only extreme ratings (5-star vs 1-star) and by adding 3-star reviews alternately to the negative group (4-5 vs 1-3) and to the positive group (3-5 vs 1-2). These networks represent comention of aspect categories within the same review and not covalence or evidence that both aspects carried the same sentiment polarity.

Because Jaccard similarity is sensitive to marginal aspect prevalence—aspects that are individually more common in 1 rating group will mechanically produce higher co-occurrence counts—we conducted a prevalence-adjusted sensitivity analysis. For each aspect pair within each rating group, we computed pointwise mutual information (PMI): PMI(A,B)=log₂(P(A∩B)/(P(A)·P(B))), where P(A) and P(B) denote each aspect’s marginal prevalence and P(A∩B) denotes their comention proportion within that rating group. Positive PMI indicates that 2 aspects co-occur more frequently than expected under independence; negative PMI indicates less frequent co-occurrence than expected. Differences between rating groups were summarized as ΔPMI=PMI(Positive) – PMI(Negative) and compared with ΔJaccard to assess whether raw co-occurrence differences persisted after prevalence adjustment.

### Ethical Considerations

This study analyzed publicly available online reviews posted on Google Maps and did not involve interaction with individuals, intervention, or access to private identifiable information. Under Taiwan’s Human Subjects Research Act (人體研究法, Article 5), research falling within the categories of exempt research announced by the competent authority does not require institutional review board review. The applicable announcement (Department of Health, Executive Yuan, now the Ministry of Health and Welfare; Announcement number 1010265075, July 5, 2012) exempts, among other categories, nonidentifiable, noninteractive, and noninterventional research conducted in public settings from which no specific individual can be identified, and research using lawfully and publicly disclosed information where the use is consistent with the purpose of that disclosure. This study meets both criteria. No attempt was made to contact reviewers or reidentify individuals. Hospital identifiers were masked in reporting to protect institutional privacy, and only short verbatim excerpts were used where necessary to minimize disclosure risk.

## Results

Results are organized by research questions: research question 1 (aspect diversity and single-topic representation), research question 2 (mixed-sentiment reviews), and research question 3 (co-occurrence networks by rating).

### Validation Results

A validation sample of 201 reviews (1407 review-aspect units) was drawn using stratified random sampling by star rating to ensure proportional representation across rating levels (each star-rating stratum differed by less than 3 percentage points from the full corpus). Two independent annotators coded each review-aspect unit on a 4-level scale (absent, negative, neutral, and positive) across 7 aspect categories, achieving substantial overall agreement (Cohen κ=0.82). The consensus outcome was designated as the gold standard for GPT validation.

Against the gold-standard annotations, GPT-4o achieved strong 4-class validation performance: accuracy=0.89, weighted *F*_1_=0.89, and Cohen κ=0.78 (95% bootstrap CI 0.74-0.81). Decomposing this into 2 tasks, aspect detection (present vs absent) yielded accuracy=0.91 and Cohen κ=0.79 (95% bootstrap CI 0.76-0.82). Per-aspect *F*_1_ ranged from 0.77 (Administrative Processes) to 0.93 (Inpatient Care), with precision consistently exceeding 0.81 across all aspects, indicating that GPT-4o rarely hallucinated nonexistent aspects (Table 3). Recall was lowest for Administrative Processes (0.67) and Surgical and Specialty Care (0.68), suggesting that prevalence estimates for these aspects may be slightly conservative. Conditional on both GPT-4o and the gold standard identifying the aspect, sentiment agreement was substantial (κ=0.87, 95% bootstrap CI 0.83-0.92; n=410 review-aspect pairs). Beyond the gold standard validation, we also examined the model-reported confidence field, which was retained as a diagnostic indicator but not used to determine the primary classifications. Confidence was high overall (mean 0.915; 99.7% of review-aspect combinations had confidence ≥0.80). Filtering out review-aspect combinations with confidence <0.80 produced nearly identical mixed-sentiment prevalence (347 vs 349 cross-aspect mixed reviews). More stringent thresholds removed substantial numbers of otherwise valid aspect mentions (eg, confidence ≥0.90 retained 8257 of 9673 review-aspect combinations and reduced cross-aspect mixed reviews to 197), so confidence was used only for sensitivity assessment rather than as an exclusion criterion.

### Quantification of Representational Compression

GPT-based ABSA produced 15,752 aspect mentions across 7 service quality dimensions, aggregating to 9673 unique review-aspect combinations (Table 2). At the review-aspect level, Service Attitude was the most frequently identified dimension (29.8% of review-aspect combinations), followed by Professional Quality (25.8%) and Administrative Processes (14.2%). Sentiment composition varied substantially across aspects. Administrative Processes was overwhelmingly negative (84.3% negative), whereas Surgical and Specialty Care was majority positive (60.9% positive). Professional Quality and Inpatient Care showed polarized distributions—both had majority-positive sentiment (57.6% and 54.0%, respectively), yet substantial negative proportions (38.6% and 38.8%)—a pattern obscured by their modestly positive mean sentiment scores (+0.20 and +0.16).

Table 4 shows the aspect diversity distribution. Of 5467 reviews, 749 (13.7%) contained no identifiable service quality aspects—typically very short reviews (median 8 characters) or off-topic content (eg, historical descriptions and lost-and-found notices). Among the remaining 4718 reviews with identifiable aspects, mean aspect diversity was 2.05 (SD 0.97; 95% bootstrap CI 2.02-2.08; median=2), and 67.4% mentioned at least 2 aspects.

**Table 4:** Aspect diversity distribution.

Aspects per review	Frequency	Values, %
0	749	13.7
1	1536	28.1
2	1866	34.1
3	958	17.5
≥4	358	6.5
Total	5467	100.0

The zero-aspect category reveals a methodological difference. Under dominant-topic assignment, LDA assigns exactly 1 topic to all 5467 reviews—including very short or off-topic content—because the model requires every document to have a topic distribution. In contrast, ABSA can recognize when a review contains no identifiable service quality content, avoiding classification of uninformative text. This distinction is relevant for interpreting representational differences between the methods.

Among reviews with at least 1 identifiable aspect (n=4718), mean aspect diversity was 2.05, whereas dominant-topic assignment represents each review by exactly 1 topic by design. Applying the compression formula defined in Methods section, (2.05–1.00)/2.05×100=51.2% (95% bootstrap CI 50.6%-51.9%), corresponding to a difference of 1.05 represented aspects per review between GPT-ABSA and dominant-topic assignment. This statistic is descriptive and partly definitional because Mean_LDA is fixed at 1.00 under dominant-topic assignment. The calculation was restricted to the 4718 reviews with at least 1 identifiable service-quality aspect to focus on reviews containing substantive service-quality content; when the 749 zero-aspect reviews were included, Mean_ABSA decreased to 1.77 (95% bootstrap CI 1.74-1.80) and the aggregate compression estimate decreased to 43.5% (95% bootstrap CI 42.5%-44.4%). At the review level, these 749 zero-aspect reviews represent negative-compression cases because LDA still assigns 1 topic whereas ABSA assigns no service-quality aspect.

### Soft-Assignment LDA Baseline

The 51.2% compression is computed against dominant-topic (argmax) assignment, a downstream simplification of LDA. Because LDA formally represents documents as topic mixtures, an additional comparison relaxed the dominant-topic constraint by retaining all topics above posterior probability thresholds (≥0.10 to ≥0.30) or the top-k topics (k=2, 3). Table 5 reports representative configurations: the original dominant-topic baseline, a conservative threshold setting (≥0.20), the threshold setting that most closely matched GPT-ABSA in mean retained-label count (≥0.15), the comparable top-k setting (top 2), and the GPT-ABSA reference. In the top-2 condition, topics with posterior probability below 0.01 were excluded, so a small subset of reviews retained only 1 topic and the mean number of topics per review was 1.968 rather than exactly 2.000. This comparison evaluates whether relaxing LDA’s dominant-topic simplification recovers the service-quality aspect structure identified by the validated GPT-ABSA pipeline, not whether GPT-ABSA constitutes a definitive ground truth for topic modeling. Table 5 summarizes the results across compression and set-level semantic alignment.

**Table 5:** Soft-assignment LDA^a^ baseline comparison (n=4718 matched reviews)^b^.

Method	Mean retained topics or aspects per review	Compression, %	Mean set Jaccard
Dominant (argmax)	1.000	51.2	0.280
Threshold ≥0.20	1.744	14.9	0.325
Threshold ≥0.15	2.016	1.7	0.334
Top 2	1.968	4.0	0.324
GPT^c^-ABSA^d^ (reference)	2.050	0.0	1.000

^a^LDA: Latent Dirichlet Allocation.

^b^For LDA rows, the mean refers to retained topic labels per review; for the GPT-ABSA reference row, it refers to GPT-derived aspect labels per review. Compression = (Mean_ABSA – Mean_LDA)/Mean_ABSA (see Methods section). Mean set Jaccard = average Jaccard similarity between each review’s soft-LDA topic set and its ABSA aspect set. LDA topics were mapped to the 7 aspect labels using the topic labels in Table 1. Top-k assignments excluded topics with posterior probability below 0.01. Additional thresholds (≥0.10, ≥0.25, and ≥0.30) and top 3 showed similar patterns; representative configurations are reported here.

^c^GPT: Generative Pretrained Transformer.

^d^ABSA: aspect-based sentiment analysis.

Soft-assignment substantially reduces count-level compression. At threshold ≥0.15, the mean number of retained LDA topics per review (2.016) nearly matches the mean number of GPT-ABSA aspects per review (2.050), reducing compression from 51.2% to 1.7%. However, count recovery did not necessarily recover the same service-quality dimensions. The soft-LDA topic set showed limited overlap with the GPT-derived aspect set (mean set Jaccard 0.334 at threshold ≥0.15). While the set-level Jaccard coefficient summarizes overall alignment, it does not reveal how individual topics map to specific aspects. An exploratory co-occurrence mapping (Table S1 in Multimedia Appendix 1) shows that across all 7 soft-assigned LDA topics, the most frequent GPT-ABSA aspect accounted for only 25.9%-37.8% of topic-aspect pairs, indicating partial semantic overlap but no clear one-to-one alignment. For example, topic 4 (Emergency Care) co-occurred most frequently with Service Attitude (29.5%) and Professional Quality (28.2%), not with its nominal label Emergency Care (10.5%).

Beyond count and semantic alignment, a third layer of representational difference is fundamental: LDA topics carry no aspect-level sentiment polarity. Even a perfectly aligned soft-LDA assignment does not distinguish whether a patient mentioned Professional Quality positively or negatively, limiting its ability to detect mixed-sentiment reviews.

### Characterization of Mixed-Sentiment Reviews

Research question 2 examines mixed evaluations—cases where patients simultaneously praise and criticize different parts of the care experience within a single review. These patterns are not directly represented by dominant-topic assignment, which assigns a single topic label without distinguishing sentiment polarity across aspects. Combining aspect identification with sentiment detection allows such trade-offs to be observed and quantified.

We distinguish 2 forms of mixed sentiment. Cross-aspect mixing refers to reviews containing at least 1 positive aspect and at least 1 negative aspect (trade-offs across dimensions). Within-aspect mixing refers to reviews containing both positive and negative opinions within the same aspect (trade-offs within a dimension).

### Prevalence and Narrative Richness

Most reviews discussed more than 1 dimension of care: 3182 of 4718 (67.4%) reviews mentioned at least 2 distinct aspects. Among these multiaspect reviews, 349 (11.0%) exhibited cross-aspect mixed sentiment—simultaneously praising certain dimensions while criticizing others. This corresponds to 7.4% of all 4718 aspect-bearing reviews. Of these 349 cross-aspect mixed reviews, 40 (11.5%) were 3-star reviews. Within-aspect mixed sentiment occurred in 322 reviews. In total, 609 reviews exhibited any form of mixed sentiment, with 62 reviews showing both cross-aspect and within-aspect mixing (ie, 609=349+322–62; these 62 hybrid reviews were included in the cross-aspect mixed group in Table 6).

**Table 6:** Cross-aspect mixed-sentiment review characteristics^a^.

Characteristic	Cross-aspect mixed-sentiment (n=349)	Cross-aspect non–mixed-sentiment (n=2833)	Statistical test
Aspect diversity	2.89 (SD 0.95)	2.52 (SD 0.74)	*U*=605,582; *P*<.001; *r*=–0.22
Review length	227.1 (SD 287.9)	141.9 (SD 182.3)	*U*=623,322; *P*<.001; *r*=–0.26
Star rating	2.87 (SD 1.67)	2.90 (SD 1.93)	*U*=486,048; *P*=.57; *r*=0.02

^a^Mann-Whitney *U* tests with rank-biserial correlation (*r*) as effect size. The non–mixed comparison group n (3182–349=2833) coincidentally equals the count of empty-text reviews excluded during data collection; the 2 figures derive from unrelated calculations.

To characterize cross-aspect mixed-sentiment reviews, we compared them with cross-aspect non–mixed-sentiment reviews (Table 6). This comparison excludes single-aspect reviews, ensuring that both groups contain reviews discussing multiple dimensions. Cross-aspect mixed-sentiment reviews were more elaborate narratives: they mentioned 15% more distinct aspects (2.89 vs 2.52; *P*<.001; rank-biserial *r*=–0.22) and were 60% longer (227 vs 142 characters; *P*<.001; *r*=–0.26) but did not differ in star rating (2.87 vs 2.90; *P*=.57).

Table 6 shows that cross-aspect mixed-sentiment reviews are structurally distinct from other multiaspect reviews: they cover more service-quality dimensions and contain more detailed narratives, yet their star ratings are indistinguishable. The similar mean star ratings do not indicate convergence on intermediate evaluations: the star ratings of these reviews were bimodal. Of the 349 cross-aspect mixed-sentiment reviews, 161 (46.1%) were rated 1-2 stars, 40 (11.5%) 3 stars, and 148 (42.4%) 4-5 stars.

### Technical-Functional Divergence

To identify which trade-offs most commonly co-occur, we analyzed cross-polarity couplings within cross-aspect mixed-sentiment reviews. Each coupling pairs a positively evaluated aspect with a negatively evaluated aspect, and we counted how many reviews contained each specific pairing. With 7 aspect categories, there are 42 possible ordered cross-polarity pairings (7 × 6, excluding same-aspect pairs). Table 7 reports the 4 most frequent couplings, ranked by review count; Table S2 in Multimedia Appendix 1 reports the full set of 42 pairings.

**Table 7:** Most frequent cross-polarity couplings within cross-aspect mixed reviews (N=349)^a^.

Rank	Positively evaluated aspect	Negatively evaluated aspect	Reviews	Pattern
1	Professional Quality	Administrative Processes	75	Technical (+) vs Functional (–)
2	Professional Quality	Service Attitude	70	Technical (+) vs Functional (–)
3	Professional Quality	Facility and Environment	69	Technical (+) vs Functional (–)
4	Service Attitude	Facility and Environment	57	Functional (+) vs Functional (–)

^a^Pairing counts are not mutually exclusive; a single review could contribute to multiple directed positive-negative pairings.

A striking pattern emerges: the 3 most frequent cross-polarity couplings all involve positive Professional Quality paired with negative functional dimensions. Out of 42 possible pairings, the top 3 share the same structure—patients praising clinical competence while criticizing operational or experiential aspects. We use the term Technical-Functional Divergence to describe this observed pattern: positive evaluations of clinical competence co-occurring with negative evaluations of operational or experiential aspects. This pattern suggests that patients may evaluate *what* is delivered (technical quality) separately from *how* it is delivered (functional quality), although this inference is drawn from textual co-occurrence rather than direct measurement of cognitive evaluation processes. Among the 349 cross-aspect mixed-sentiment reviews, 215 (61.6%) exhibited Technical-Functional Divergence. An illustrative excerpt is as follows:

The dentist was professional and patient, but the staff member taking X-rays had an extremely bad attitude—almost swearing. If you do not want to do the job, go home; do not hold others back.

This example illustrates Technical-Functional Divergence: the patient praises clinical competence (technical quality) while criticizing staff behavior (functional quality), articulating these dimensions as distinct evaluative targets within the same review.

### Within-Aspect Heterogeneity

Within-aspect mixed sentiment occurred in 322 reviews. Table 8 reports within-aspect mixing by aspect category. Service Attitude showed the highest mixing rate (4.7%), followed by Facility and Environment (4.2%) and Professional Quality (4.0%). Administrative Processes showed the lowest rate (1.3%), likely because administrative encounters are more homogeneous than clinical or interpersonal encounters.

**Table 8:** Within-aspect mixed sentiment by aspect category^a^.

Aspect category	Within-aspect mixed reviews	Reviews mentioning aspect	Mixing rate, %
Service Attitude	136	2884	4.7
Facility and Environment	37	877	4.2
Professional Quality	99	2495	4.0
Inpatient Care	25	842	3.0
Emergency Care	18	626	2.9
Surgical and Specialty Care	15	578	2.6
Administrative Processes	18	1371	1.3

^a^Each row counts unique reviews exhibiting within-aspect mixing for that aspect. A single review may appear in multiple rows if it exhibits within-aspect mixing in more than 1 aspect category.

The high within-aspect mixing rate for Service Attitude reflects the heterogeneous nature of interpersonal encounters during a hospital visit. Patients interact with multiple staff roles—physicians, nurses, administrative personnel, technicians, and volunteers—each of whom may elicit different evaluations. An illustrative example:

The nurses and doctors were all patient and had good attitudes...but the pharmacist had a terrible tone.

Together, these findings suggest that mixed-sentiment reviews constitute a distinct subset of patient feedback that dominant-topic assignment does not directly capture.

### Aspect Co-Occurrence Profiles by Rating

#### Operationalization

To examine whether high- and low-rated reviews organize aspect mentions differently, we constructed rating-stratified aspect co-occurrence networks. Reviews rated 4-5 stars were defined as positive reviews (n=1961) and reviews rated 1-2 stars as negative reviews (n=2581); 3-star reviews were treated as neutral and excluded. Within each network, aspects were connected using the Jaccard similarity coefficient, J(A,B)=|A∩B|/|A∪B|, where A and B denote the sets of reviews that mention each aspect. Edges therefore represent comention of aspect categories within reviews and not covalence or evidence that both aspects were evaluated with the same polarity.

#### Rating-Stratified Comention Patterns

We compared co-occurrence strength across all 21 aspect pairs between positive and negative reviews using the bootstrap and permutation procedures described in Methods section. Fourteen of 21 pairs remained significant after false discovery rate correction (*q*<.05; Table S3 in Multimedia Appendix 1 reports the full set).

Table 9 highlights a shared core pairing and the pairings with the largest raw Jaccard differences. Specifically, we first report the single strongest pairing in both groups (highest Jaccard). We then report pairings with the largest positive and negative Δ values after excluding this core pairing.

**Table 9:** Aspect co-occurrence patterns by rating group (raw Jaccard)^a^.

Aspect pair			Positive		Negative		Ratio		Δ		95% CI		FDR *P* value *q*^b^
Core coupling (both groups)													
	Professional Quality ↔ Service Attitude	0.509		0.357		1.4		+0.152		0.120 to 0.185		<.001	
Higher raw Jaccard in positive reviews													
	Inpatient Care ↔ Surgical and Specialty Care	0.316		0.123		2.6		+0.193		0.147 to 0.241		<.001	
	Professional Quality ↔ Surgical and Specialty Care	0.210		0.089		2.4		+0.121		0.095 to 0.147		<.001	
	Inpatient Care ↔ Professional Quality	0.207		0.095		2.2		+0.112		0.085 to 0.138		<.001	
Higher raw Jaccard in negative reviews													
	Administrative Processes ↔ Service Attitude	0.110		0.255		2.3		–0.145		–0.170 to –0.120		<.001	
	Administrative Processes ↔ Professional Quality	0.110		0.231		2.1		–0.121		–0.147 to –0.096		<.001	
	Emergency Care ↔ Service Attitude	0.098		0.174		1.8		–0.076		–0.100 to –0.052		<.001	

^a^Positive reviews are rated 4-5 stars (n=1961) and negative reviews are rated 1-2 stars (n=2581). Values represent Jaccard co-occurrence strength. Ratio = higher group Jaccard/lower group Jaccard. Δ = Jaccard(Positive) – Jaccard(Negative). 95% CI from 2000 bootstrap resamples. FDR q from 5000 label-permutation tests with Benjamini-Hochberg correction across 21 pairs.

^b^FDR *q*: false discovery rate–adjusted *P* value (*q* value) obtained using the Benjamini-Hochberg procedure.

The raw Jaccard profiles in Table 9 show that positive and negative reviews differ in which aspects are comentioned. Professional Quality ↔ Service Attitude is the strongest pairing in both groups (Jaccard = 0.509 in positive, 0.357 in negative), indicating that patients consistently discuss clinical competence alongside interpersonal quality regardless of overall satisfaction. Among the remaining pairs, clinical aspects (Professional Quality, Inpatient Care, and Surgical and Specialty Care) show higher raw co-occurrence in positive reviews, whereas operational aspects (Administrative Processes and Service Attitude) show higher raw co-occurrence in negative reviews.

### Prevalence-Adjusted Sensitivity Analysis

However, marginal aspect prevalence differs substantially between rating groups (Table 10). For example, Surgical and Specialty Care is mentioned in 19.0% of positive reviews but only in 7.3% of negative reviews (ratio 2.6), while Administrative Processes appears in 12.7% of positive reviews versus 40.8% of negative reviews (ratio 0.3). These prevalence differences can inflate or deflate raw Jaccard values independently of whether the aspects genuinely tend to co-occur.

**Table 10:** Marginal aspect prevalence by rating group.

Aspect	Positive, %	Negative, %	Ratio
Professional Quality	70.4	40.6	1.73
Service Attitude	62.2	61.3	1.01
Inpatient Care	24.9	12.5	1.99
Facility and Environment	19.2	16.9	1.14
Surgical and Specialty Care	19.0	7.3	2.61
Administrative Processes	12.7	40.8	0.31
Emergency Care	9.8	16.3	0.60

To assess whether the raw Jaccard differences reflected genuine co-occurrence tendencies beyond base rates, we computed PMI within each rating group (Table 11). PMI values near zero indicate co-occurrence consistent with marginal prevalences; positive values indicate excess comention; negative values indicate less comention than expected.

**Table 11:** Prevalence-adjusted co-occurrence: selected pairs^a^.

Aspect pair	ΔJaccard	PMI^b^ (positive)	PMI (negative)	ΔPMI
Inpatient Care ↔ Surgical and Specialty Care	+0.193	+1.15	+1.25	–0.10
Professional Quality ↔ Service Attitude	+0.152	+0.03	+0.11	–0.07
Administrative Processes ↔ Service Attitude	–0.145	–0.10	–0.27	+0.17
Administrative Processes ↔ Professional Quality	–0.121	–0.13	–0.12	–0.01
Professional Quality ↔ Surgical and Specialty Care	+0.121	+0.21	+0.40	–0.19
Inpatient Care ↔ Professional Quality	+0.112	–0.10	–0.14	+0.04
Emergency Care ↔ Service Attitude	–0.076	+0.08	+0.20	–0.12

^a^PMI = log₂(P(A∩B)/(P(A)·P(B))) computed within each rating group. ΔPMI = PMI(positive) – PMI(negative). Full 21-pair table is shown in Table S4 in Multimedia Appendix 1.

^b^PMI: pointwise mutual information.

The prevalence-adjusted analysis reveals that several large raw Jaccard differences are substantially driven by differences in marginal aspect prevalence rather than by differential co-occurrence tendencies. Across all 21 pairs, the direction of ΔJaccard and ΔPMI agreed in only 13 of 21 cases (Spearman ρ=0.08; *P*=.74). Three patterns merit attention:

1. Inpatient Care ↔ Surgical and Specialty Care showed the largest ΔJaccard (+0.193), but both rating groups exhibited similar PMI values (positive: +1.15; negative: +1.25; ΔPMI=–0.10). This indicates that the 2 aspects co-occur more than expected in both groups; the Jaccard difference is primarily attributable to the higher individual prevalence of both aspects in positive reviews than in negative reviews.
2. Professional Quality ↔ Service Attitude—the strongest comention pair in both groups—showed PMI values near zero in both (positive: +0.03; negative: +0.11), meaning that this comention is almost entirely explained by the high marginal prevalence of both aspects rather than by a specific tendency for them to appear together.
3. Administrative Processes ↔ Service Attitude, the pair with the largest negative ΔJaccard (–0.145), showed a ΔPMI of +0.17, reversing direction. After adjusting for Administrative Processes’ much higher base rate in negative reviews (40.8% vs 12.7%), both groups showed negative PMI values (positive: –0.10; negative: –0.27), indicating less comention than expected from their marginal prevalences.

As a robustness check, we repeated the raw Jaccard analysis using only extreme ratings (5-star vs 1-star). Co-occurrence profiles were highly consistent with the 4-5 vs 1-2 grouping (Spearman ρ=0.990 for positive and 0.991 for negative; mean absolute Jaccard difference <0.01 for both), and the top 5 pairs were unchanged—supporting the stability of the raw comention profiles, although not resolving the prevalence confound. We also assessed whether excluding 3-star reviews shifted the network results. Adding the 176 3-star aspect-bearing reviews to the negative group (4-5 vs 1-3) yielded a ΔJaccard vector nearly identical to the original 4-5 vs 1-2 comparison (Spearman ρ=1.000; mean absolute Δ change=0.002; maximum change=0.005). Adding them to the positive group (3-5 vs 1-2) produced the same conclusion (Spearman ρ=0.999; mean absolute Δ change=0.003; maximum change=0.012).

## Discussion

### Principal Results

This study compared GPT-based ABSA with LDA topic modeling on patient reviews from 24 medical centers in Taiwan in a controlled but information-asymmetric design, examining what additional information ABSA provides beyond dominant-topic assignment. The findings indicate 3 main representational differences.

First, by representing each aspect-bearing review with a single topic, dominant-topic assignment produced a descriptive 51.2% representational compression estimate relative to the GPT-derived aspect structure. Relaxing LDA to soft assignment (retaining all topics above a probability threshold) reduced count-level compression to 1.7%, but semantic alignment with the ABSA aspect structure remained limited (mean set Jaccard=0.334). In addition, LDA topics do not directly encode sentiment polarity without additional sentiment modeling, limiting their ability to identify mixed-sentiment patterns in this analysis.

Second, 11.0% of multiaspect reviews contained cross-aspect mixed sentiment. This finding is consistent with the evaluative space model, which treats positive and negative appraisals as potentially coactivated rather than mutually exclusive [7]. Compared with other multiaspect reviews lacking mixed sentiment, these reviews covered 15% more distinct aspects and were 60% longer, yet their star ratings were statistically indistinguishable from those of nonmixed reviews. Among 42 possible cross-polarity pairings, the 3 most frequent couplings shared the same structure: positive Professional Quality paired with negative functional dimensions (Administrative Processes, Service Attitude, or Facility and Environment). Technical-Functional Divergence, as defined in the Methods section, appeared in 61.6% of all cross-aspect mixed-sentiment reviews, suggesting that patients may evaluate clinical competence separately from service delivery within the same encounter. Additionally, within-aspect mixed sentiment occurred in 322 reviews. Service Attitude showed the highest mixing rate (4.7%), which may reflect heterogeneous interactions across different staff roles.

Third, positive and negative reviews exhibited different comention profiles: the raw Jaccard networks showed that clinical aspects (Professional Quality, Inpatient Care, and Surgical and Specialty Care) were comentioned more frequently in positive reviews, whereas operational aspects (Administrative Processes and Service Attitude) were comentioned more frequently in negative reviews. Professional Quality ↔ Service Attitude was the strongest pairing in both groups, suggesting that patients consistently discuss clinical competence alongside interpersonal quality regardless of satisfaction level.

However, prevalence-adjusted analysis using PMI showed that these raw comention differences were substantially driven by differences in marginal aspect prevalence between rating groups rather than by differential co-occurrence tendencies. For example, Inpatient Care and Surgical and Specialty Care are each mentioned roughly twice as often in positive reviews as in negative reviews; after adjusting for these base rates, their PMI values were similar in the positive and negative groups (+1.15 vs +1.25). Across all 21 pairs, ΔJaccard and ΔPMI showed near-zero rank correlation (Spearman ρ=0.08). These results indicate that the rating-stratified comention profiles primarily reflect *which* aspects patients discuss in positive versus negative reviews (compositional differences) rather than stable co-occurrence tendencies that operate independently of base rates (associative differences).

These compositional differences are nonetheless informative for practice. The observation that positive reviews are more likely to mention clinical aspects while negative reviews disproportionately feature operational aspects is consistent with prior work distinguishing technical quality (what is delivered) from functional quality (how it is delivered) [27]. For hospital quality monitoring, clinical and operational signals cluster in different rating strata; dashboards designed to separate these streams may therefore help organize feedback more effectively, although the clustering partly reflects compositional rather than associative differences. These interpretations are based on observational comention patterns and should be treated as descriptive rather than causal; future research using experimental or longitudinal designs could test the proposed mechanisms more directly.

### Limitations

This study has several limitations. All findings are derived from publicly available Google Maps reviews rather than structured surveys or controlled experiments; accordingly, the results reflect patterns in voluntarily posted narratives and should be interpreted as observational associations rather than causal relationships. First, online reviews are subject to selection bias and likely overrepresent extreme experiences. Second, the dataset is geographically and platform-specific (Taiwan medical centers on Google Maps); cultural norms and platform affordances may influence both what patients report and how they express evaluations. Third, ABSA performance depends on prompt specification and model behavior. Although we iteratively refined prompts during development and achieved κ=0.78 against the consensus outcome through structured outputs and systematic validation, we did not conduct a formal prompt sensitivity analysis (eg, systematically varying instruction phrasing or example selection). Results may therefore be sensitive to specific prompt design choices, and residual misclassification cannot be ruled out. Specifically, because aspect detection recall ranged from 0.67 to 0.90 across aspects (Table 3), some mixed-sentiment reviews may have been classified as pure sentiment if GPT-4o missed the only positive or negative aspect in a review. The recall-gap sensitivity analysis described in Methods section yielded an observed cross-aspect mixed-sentiment prevalence of 7.4% of all aspect-bearing reviews (equivalent to 11.0% of multiaspect reviews); after per-aspect recall correction, the estimate increased to 10.5% and, under a conservative recall assumption (*r*=0.675), to an upper bound of 13.9% (both expressed as proportions of all aspect-bearing reviews). Thus, the observed prevalence may be conservative, but the core finding remains unchanged: a nontrivial subset of reviews contains evaluative tensions not captured by dominant-topic representations. More broadly, future work on LLM-based text analysis in health care could benefit from retrieval-grounded evaluation frameworks that incorporate source-linked verification [28] and from formal hallucination detection mechanisms that decompose data-driven and reasoning-driven error sources [26].

Fourth, the Technical-Functional Divergence pattern observed in this study may vary across health care contexts. Factors such as system capacity, cultural expectations, and service models may influence which functional dimensions are most susceptible to negative evaluation. Cross-national replication would help establish the boundary conditions under which this divergence pattern emerges. The growing application of LLM-based methods to analyze health-related discourse on social media [29] further underscores the need for cross-context validation of text-analytic frameworks.

Fifth, the confidence score returned by GPT-4o was a self-reported diagnostic indicator rather than an externally validated probability of correctness. We therefore did not use it as a primary exclusion criterion. A threshold sensitivity check showed that a moderate threshold (confidence ≥0.80) produced nearly unchanged mixed-sentiment prevalence, whereas stricter thresholds primarily reduced aspect coverage. Sixth, LDA results are sensitive to preprocessing decisions. Different filtering thresholds (eg, minimum token counts and character length cutoffs) can substantially alter topic structures from the same source data. The LDA side of our comparison is therefore contingent on the specific preprocessing choices described in Methods section, and different choices could yield a different topic structure and hence a different point of comparison. Seventh, our comparison was intentionally controlled but information-asymmetric: GPT-based ABSA used a domain-specific taxonomy, category definitions, and worked examples, whereas LDA was estimated in an unsupervised manner. This design reflects a practical trade-off between guided diagnostic assessment and unsupervised exploratory discovery, but it is not a level comparison between methods given equivalent input information. Future studies could implement more information-symmetric methodological foils, such as joint sentiment-topic models or BERTopic-based pipelines, to examine whether similar aspect-level evaluative structures emerge under alternative modeling assumptions.

Eighth, the primary ABSA extraction used a single LLM (GPT-4o, snapshot gpt-4o-2024-08-06). To assess model dependence, we repeated the extraction on the 201 validation reviews using 2 alternative frontier models from different providers: DeepSeek-V4-Flash nonthinking mode (DeepSeek; accessed through the legacy API identifier deepseek-chat) and Claude Sonnet 4.6 (Anthropic), applying the identical prompt template. Against the same gold standard, DeepSeek-V4-Flash achieved 4-class κ=0.77 and Claude Sonnet 4.6 achieved κ=0.76, both comparable with GPT-4o (κ=0.78). Conditional sentiment agreement was high for all 3 models (GPT-4o: κ=0.87; DeepSeek-V4-Flash: κ=0.88; and Claude Sonnet 4.6: κ=0.89). Intermodel agreement was also substantial (GPT-4o vs DeepSeek-V4-Flash: κ=0.82; GPT-4o vs Claude Sonnet 4.6: κ=0.78). These results suggest that the aspect-level findings are not critically dependent on the specific model used, although model-specific differences may be more pronounced for implicit or sarcastic expressions.

Ninth, the co-occurrence network analysis (research question 3) excluded 3-star reviews (176 reviews; 3.7% of the 4718 reviews with identifiable aspects) to create distinct positive and negative groups. Of the 349 cross-aspect mixed reviews, 40 (11.5%) fell in this 3-star band. These neutral reviews may exhibit different co-occurrence patterns; however, robustness checks using only extreme ratings (5-star vs 1-star) and adding 3-star reviews alternately to the positive or negative group produced highly consistent results (Spearman ρ≥0.999 for ΔJaccard vectors), suggesting that the specific star-rating boundary used to define positive and negative groups does not substantially alter the raw comention profiles. Tenth, the prevalence-adjusted analysis (PMI) showed that several raw Jaccard differences between rating groups were attributable to marginal aspect prevalence rather than differential co-occurrence tendencies. The research question 3 comention profiles should therefore be interpreted as compositional—reflecting which aspects patients discuss in positive versus negative reviews—rather than as evidence of prevalence-independent associative patterns.

### Comparison With Prior Work

Recent studies have validated GPT-based ABSA for service quality analysis. Hsueh and Hsu [30] achieved 89% accuracy (κ=0.76) analyzing British Museum reviews, while Li et al [24] demonstrated scalability with 94.4% precision on 504,198 Chinese patient reviews. Our validation metrics (accuracy=0.89; κ=0.78) are consistent with prior evidence of GPT-based ABSA performance across service domains.

Prior health care and user-review studies have used LDA primarily as an exploratory tool for identifying broad thematic structures in patient-generated text [10-13]. This approach is especially valuable when aspect categories are unknown, although applied interpretations often summarize each document by its dominant topic. Our findings extend this literature by quantifying the representational implications of this common simplification: dominant-topic assignment produced a descriptive 51.2% compression estimate relative to the GPT-derived aspect structure. A soft-assignment baseline that allowed multiple topics per review substantially reduced the count-level gap, suggesting that LDA can recover more multitopic structure when interpreted probabilistically; however, diagnostic service-quality assessment can benefit from explicit aspect and sentiment labels.

The mixed-sentiment findings also extend work on evaluative space and sentiment ambivalence. Prior theory argues that positive and negative appraisals can be coactivated rather than treated as opposite ends of a single continuum [7], and recent ABSA research has operationalized aspect-level sentiment ambivalence in tourism reviews [8]. Direct prevalence comparisons are difficult because studies differ in domain, unit of analysis, and operational definition. Our study extends this literature to health care reviews by showing that mixed sentiment can be localized to specific service-quality dimensions within the same patient narrative. In particular, the Technical-Functional Divergence pattern suggests that mixed sentiment may be patterned rather than random: patients may praise clinical competence while criticizing operational or experiential aspects of care.

The comention findings connect service-quality theory with network-based review analysis. Prior service-quality theory distinguishes technical quality (what is delivered) from functional quality (how it is delivered) [27], while negativity bias research suggests that negative information may carry disproportionate weight in evaluative judgment [31]. Our rating-stratified comention analysis showed that positive and negative reviews differed in which aspects were discussed: clinical dimensions appeared more frequently in positive reviews, whereas operational dimensions featured more prominently in negative reviews. However, prevalence-adjusted analysis (PMI) revealed that these raw comention differences were largely attributable to marginal prevalence shifts rather than differential co-occurrence tendencies. This finding underscores the importance of distinguishing compositional differences from associative differences in co-occurrence network analyses of patient reviews.

### Conclusions

In this corpus, dominant-topic assignment compressed multiaspect patient feedback; soft-assignment LDA recovered topic counts but did not restore semantic alignment or aspect-level sentiment polarity. A nontrivial subset of reviews exhibited cross-aspect mixed sentiment—most commonly praising clinical competence while criticizing functional dimensions—and positive and negative reviews discussed different constellations of quality dimensions.

Technical-Functional Divergence at the individual-review level, compositional differences in aspect comention across rating strata, and within-aspect sentiment heterogeneity each point to evaluative complexity that dominant-topic representations are not designed to capture. The prevalence-adjusted analysis further showed that raw comention differences between positive and negative reviews primarily reflected which aspects patients discussed rather than differential associative tendencies, underscoring the need to distinguish compositional from associative patterns in co-occurrence analyses.

For practice, quality-monitoring systems could usefully distinguish clinical signals from operational signals and jointly track co-occurring operational complaints rather than treating them as independent departmental issues. Mixed-sentiment reviews, which are longer and more aspect-diverse and yet produce indistinguishable star ratings, may contain diagnostically relevant feedback that aggregate metrics may not make visible.

This study does not argue against topic modeling entirely. LDA remains valuable for exploratory discovery when aspect categories are unknown. These findings support a complementary approach: use topic modeling for initial exploration and ABSA for diagnostic assessment when multidimensional quality evaluation is required.

The observational patterns also suggest directions for confirmatory research. Whether patients genuinely evaluate clinical and functional quality as independent dimensions could be tested through experimental or survey-based designs rather than inferred from textual co-occurrence. Similarly, whether flagging mixed-sentiment reviews in quality dashboards improves managerial decision-making warrants intervention studies. Additionally, the persistent coupling of Professional Quality and Service Attitude observed in this study raises a question that warrants further investigation: whether interpersonal quality functions not merely as another service dimension but as a trust-mediated channel through which patients infer clinical competence when they cannot directly evaluate technical quality.

## Data Availability

Prompt templates and analysis scripts are deposited in a public GitHub repository [32] and permanently archived on Zenodo [33]. Raw Google Reviews cannot be redistributed due to Google Maps platform terms of service; the corresponding author will provide derived aggregate data upon reasonable request to facilitate verification. Multimedia Appendix 1 provides the full aspect-based sentiment analysis prompt and an example JavaScript Object Notation output, the Latent Dirichlet Allocation coherence curve for topic number selection (Figure S1), the exploratory soft-LDA topic-to-aspect mapping (Table S1), the full directed cross-polarity coupling table (Table S2), the full aspect-pair Jaccard co-occurrence inference table (Table S3), and the full prevalence-adjusted co-occurrence analysis (Table S4).

## Appendix

Multimedia Appendix 1 Generative Pretrained Transformer–based aspect-level sentiment analysis prompt design (Chinese original and English translation), example JavaScript Object Notation output, Latent Dirichlet Allocation (LDA) coherence curve for topic number selection, exploratory soft-LDA topic-to-aspect-based sentiment analysis aspect mapping, full directed cross-polarity coupling table (42 pairings), and full aspect-pair Jaccard co-occurrence inference table (21 pairs).

## Abbreviations

ABSA: aspect-based sentiment analysis
GPT: Generative Pretrained Transformer
LDA: Latent Dirichlet Allocation
LLM: large language model
PMI: pointwise mutual information
SERVQUAL: Service Quality
