# Supplementary material for: What Single-Topic Summaries Miss in Hospital Reviews—Aspect-Level Evaluative Structure Using Generative Pretrained Transformer–Based Sentiment Analysis: Content Analysis
Source: J Med Internet Res. 2026 Sep 14;28:e92325. doi: 10.2196/92325 (PMC13620426; doi:10.2196/92325)
Supplement: Supplementary file 1 [file jmir_v28i1e92325_app1.docx]

# Multimedia Appendix 1

## Complete ABSA Prompt Template

The following prompt was used with GPT-4o (snapshot gpt-4o-2024-08-06; temperature = 0; API call date: November 30, 2025) to extract aspect-sentiment pairs from each review. The prompt was written in Traditional Chinese to match the source language of the reviews. Section A presents the original Chinese prompt; Section B provides a parallel English translation for international readers.

In both versions, {review_text} is a template placeholder where each individual review was inserted before submission to the API.

### A. Original Prompt (Traditional Chinese)

你是一位專業的醫療服務品質分析專家。請分析以下台灣醫院的病患評論，識別其中提到的醫療服務品質構面（aspects）及其情感傾向。

**任務說明**

請根據評論內容，識別出所有提到的品質構面，並進行情感分析。每個構面都需要標註：

1. **aspect_category**: 品質構面類別（從以下 7 個預定義類別中選擇）
2. **aspect_term**: 具體提及的面向詞彙（例如：「醫師態度」、「等待時間」）
3. **opinion**: 原文摘錄（不要改寫，保留原始文字）
4. **sentiment**: 情感極性（positive / negative / neutral）
5. **sentiment_score**: 情感分數（**只能是 -1, 0, 或 +1**）
   - positive（正面）→ +1
   - neutral（中立）→ 0
   - negative（負面）→ -1
6. **confidence**: 信心分數（0.0 到 1.0，你對這個判斷的確定程度）

**7 個預定義品質構面**

1. **Emergency Care（急診醫療服務）**

- 定義: 急診等待時間、急診醫療品質、急診流程、急診設備
- 範例詞彙: 急診、等很久、急診室、急救、緊急處理
- 範例句子: 「急診等了3小時才看到醫生」、「急診醫生很專業」

2. **Professional Quality（醫療專業品質）**

- 定義: 醫師專業能力、診斷準確性、治療效果、醫療技術、檢查品質
- 範例詞彙: 醫師、專業、檢查、診斷、治療、外科、內科
- 範例句子: 「醫師很耐心解釋病情」、「檢查很仔細」

3. **Service Attitude（服務態度問題）**

- 定義: 醫護人員態度、溝通方式、同理心、尊重病患
- 範例詞彙: 態度、不耐煩、冷漠、親切、有禮貌、沒同理心
- 範例句子: 「護理師態度很差」、「醫生很有耐心」

4. **Administrative Processes（掛號繳費流程）**

- 定義: 掛號流程、看診流程、等待時間（非急診）、批價繳費、報到程序
- 範例詞彙: 掛號、等待、看診、預約、批價、繳費、報到
- 範例句子: 「掛號等很久」、「看診流程混亂」

5. **Facility & Environment（設施環境品質）**

- 定義: 醫院環境、設施狀況、清潔度、空間規劃、交通便利性
- 範例詞彙: 環境、設施、乾淨、舒適、停車、老舊
- 範例句子: 「醫院環境很乾淨」、「設備老舊」

6. **Surgical & Specialty Care（手術專科照護）**

- 定義: 手術品質、術前術後照護、專科醫療、複雜治療
- 範例詞彙: 手術、開刀、術後、專科、治療、復健
- 範例句子: 「手術很成功」、「術後照護不佳」

7. **Inpatient Care（住院照護經驗）**

- 定義: 住院服務、病房環境、護理照護、住院流程、出院流程
- 範例詞彙: 住院、病房、護理師、照護、出院
- 範例句子: 「住院期間護理師很用心」、「病房環境舒適」

**輸出格式（JSON）**

請以 JSON 格式輸出，包含一個 aspects 陣列，每個元素代表一個識別出的品質構面。

範例輸出:

```
{
  "aspects": [
    {
      "aspect_category": "Emergency Care",
      "aspect_term": "等待時間",
      "opinion": "急診等了3小時才看到醫生",
      "sentiment": "negative",
      "sentiment_score": -1,
      "confidence": 0.95
    },
    {
      "aspect_category": "Emergency Care",
      "aspect_term": "醫生專業",
      "opinion": "急診醫生處理很專業",
      "sentiment": "positive",
      "sentiment_score": 1,
      "confidence": 0.9
    },
    {
      "aspect_category": "Professional Quality",
      "aspect_term": "檢查仔細",
      "opinion": "醫生很仔細檢查",
      "sentiment": "positive",
      "sentiment_score": 1,
      "confidence": 0.9
    }
  ]
}
```

**重要規則**

1. 一個評論可以包含多個構面（例如：同時提到 Emergency Care + Professional Quality）
2. 一個構面可以重複出現多次（例如：「急診等很久」和「急診醫生態度差」都屬於 Emergency Care，但分別輸出兩筆）
3. 每個細項獨立評分（即使同屬一個構面，每個 aspect_term 都要獨立標註情感）
4. 情感極性可以不同（同一構面內可以有正面和負面的細項，例如：急診等待時間差但醫生專業）
5. opinion 必須是原文摘錄（不要改寫或翻譯，保留病患的原始用詞）
6. sentiment_score 只能是 -1, 0, 或 +1（正面=+1，中立=0，負面=-1）
7. 所有 aspect_category 必須從上述 7 個類別中選擇
8. 如果評論為空或無法識別任何構面，回傳空陣列 {"aspects": []}
9. 結合上下文判斷情感：
   - 請仔細閱讀整則評論的上下文
   - 注意前後句的邏輯關係
   - 如果正面詞彙後接批評或抱怨，該正面詞彙可能是諷刺或反諷
10. 台灣口語的諷刺用法：
    - “有夠棒的”、“真棒”、“超好的”、“讚啦” 等過度正面詞彙
    - 若後續內容是抱怨、問題描述，或評論整體語氣負面，這些詞彙通常是諷刺
    - 請依據整體語氣和後續內容判斷真實情感

**病患評論**

{review_text}

請分析上述評論並以 JSON 格式輸出結果。

### B. English Translation

You are a professional healthcare service quality analyst. Please analyze the following patient review from a Taiwanese hospital. Identify all healthcare service quality dimensions (aspects) mentioned and their sentiment orientation.

**Task Description**

Based on the review content, identify all mentioned quality dimensions and perform sentiment analysis. Each dimension must be annotated with:

1. **aspect_category**: Quality dimension category (select from the 7 predefined categories below)
2. **aspect_term**: The specific term mentioned (eg, “doctor’s attitude,” “waiting time”)
3. **opinion**: Verbatim excerpt (do not paraphrase; preserve the original wording)
4. **sentiment**: Sentiment polarity (positive / negative / neutral)
5. **sentiment_score**: Sentiment score (**must be −1, 0, or +1 only**)
   - positive → +1
   - neutral → 0
   - negative → −1
6. **confidence**: Confidence score (0.0 to 1.0, your certainty about the judgment)

**Seven Predefined Quality Dimensions**

1. **Emergency Care**

- Definition: Emergency department waiting time, emergency medical quality, emergency procedures, emergency equipment
- Example terms: emergency department, long wait, emergency room, first aid, urgent treatment
- Example sentences: “Waited 3 hours in the ER before seeing a doctor”; “The ER doctor was very professional”

2. **Professional Quality**

- Definition: Physician expertise, diagnostic accuracy, treatment effectiveness, medical technology, examination quality
- Example terms: physician, professional, examination, diagnosis, treatment, surgery, internal medicine
- Example sentences: “The doctor patiently explained the condition”; “The examination was thorough”

3. **Service Attitude**

- Definition: Healthcare staff attitude, communication style, empathy, respect for patients
- Example terms: attitude, impatient, indifferent, friendly, polite, lacking empathy
- Example sentences: “The nurse had a terrible attitude”; “The doctor was very patient”

4. **Administrative Processes**

- Definition: Registration process, consultation flow, waiting time (non-emergency), billing and payment, check-in procedures
- Example terms: registration, waiting, consultation, appointment, billing, payment, check-in
- Example sentences: “Registration took a long time”; “The consultation process was chaotic”

5. **Facility & Environment**

- Definition: Hospital environment, facility conditions, cleanliness, space planning, transportation accessibility
- Example terms: environment, facilities, clean, comfortable, parking, outdated
- Example sentences: “The hospital environment was very clean”; “The equipment was outdated”

6. **Surgical & Specialty Care**

- Definition: Surgical quality, pre- and post-operative care, specialty medical care, complex treatments
- Example terms: surgery, operation, post-operative, specialty, treatment, rehabilitation
- Example sentences: “The surgery was successful”; “Post-operative care was inadequate”

7. **Inpatient Care**

- Definition: Inpatient services, ward environment, nursing care, admission process, discharge process
- Example terms: hospitalization, ward, nurse, care, discharge
- Example sentences: “The nurses were very attentive during hospitalization”; “The ward environment was comfortable”

**Output Format (JSON)**

Output in JSON format with an aspects array, where each element represents one identified quality dimension.

Example output:

```
{
  "aspects": [
    {
      "aspect_category": "Emergency Care",
      "aspect_term": "waiting time",
      "opinion": "Waited 3 hours in the ER before seeing a doctor",
      "sentiment": "negative",
      "sentiment_score": -1,
      "confidence": 0.95
    },
    {
      "aspect_category": "Emergency Care",
      "aspect_term": "doctor expertise",
      "opinion": "The ER doctor handled it very professionally",
      "sentiment": "positive",
      "sentiment_score": 1,
      "confidence": 0.9
    },
    {
      "aspect_category": "Professional Quality",
      "aspect_term": "thorough examination",
      "opinion": "The doctor examined very carefully",
      "sentiment": "positive",
      "sentiment_score": 1,
      "confidence": 0.9
    }
  ]
}
```

**Critical Rules**

1. A single review may contain multiple dimensions (eg, both Emergency Care and Professional Quality).
2. The same dimension may appear multiple times (eg, “long ER wait” and “rude ER doctor” both belong to Emergency Care but are output as separate entries).
3. Each entry is scored independently (even entries under the same dimension receive individual sentiment annotations).
4. Sentiment polarity may differ within the same dimension (eg, poor ER waiting time but professional doctor).
5. The opinion field must be a verbatim excerpt (do not paraphrase or translate; preserve the patient’s original wording).
6. sentiment_score must be −1, 0, or +1 only (positive = +1, neutral = 0, negative = −1).
7. All aspect_category values must be selected from the 7 categories listed above.
8. If the review is empty or no dimension can be identified, return an empty array: {"aspects": []}.
9. Use contextual cues to determine sentiment:
   - Read the full context of the review carefully.
   - Pay attention to logical relationships between sentences.
   - If positive wording is followed by complaints or criticism, the positive language may be sarcastic or ironic.
10. Sarcasm in Taiwanese colloquial expressions:
    - Phrases such as “有夠棒的” (how wonderful), “真棒” (really great), “超好的” (super good), “讚啦” (awesome) are exaggeratedly positive expressions.
    - If subsequent content contains complaints, problem descriptions, or an overall negative tone, these expressions are typically sarcastic.
    - Determine true sentiment based on overall tone and subsequent content.

**Patient Review**

{review_text}

Please analyze the above review and output the results in JSON format.

## Figure S1. LDA Coherence Curve for Topic Number (K) Selection

LDA coherence across candidate K

**Figure S1.** C_v coherence scores for LDA models with K = 2–15 topics. The red dashed line indicates the selected K = 7. Coherence was highest at K = 2 (C_v = .518), reflecting overly broad clusters that collapse multiple service quality dimensions. For K ≥ 5, coherence entered a plateau (range: .426–.491). An isolated spike at K = 12 (C_v = .491) was not sustained at adjacent values (K = 11: .426; K = 13: .457), suggesting an unstable solution. Within this plateau, K = 7 represented a local maximum (C_v = .486) and yielded seven semantically coherent topics corresponding to distinct healthcare service quality dimensions.

## Table S1. Exploratory Soft-LDA Topic-to-GPT-ABSA Aspect Co-occurrence Mapping at Threshold ≥0.15

| **Soft-assigned LDA topic** | **Selected reviews, n** | **Total topic-aspect pairs** | **Primary ABSA aspect** | **Primary share of pairs (%)** | **Secondary ABSA aspect** | **Secondary share of pairs (%)** |
| --- | --- | --- | --- | --- | --- | --- |
| T1 Service Attitude | 1,797 | 3,498 | Service Attitude | 34.5 | Administrative Processes | 18.4 |
| T2 Administrative Processes | 1,413 | 2,793 | Administrative Processes | 31.0 | Service Attitude | 26.4 |
| T3 Professional Quality | 845 | 1,727 | Professional Quality | 37.8 | Service Attitude | 28.1 |
| T4 Emergency Care | 1,684 | 3,904 | Service Attitude | 29.5 | Professional Quality | 28.2 |
| T5 Inpatient Care | 1,938 | 4,332 | Service Attitude | 30.3 | Professional Quality | 27.6 |
| T6 Surgical & Specialty Care | 865 | 1,999 | Professional Quality | 28.1 | Service Attitude | 25.4 |
| T7 Facility & Environment | 971 | 1,953 | Service Attitude | 25.9 | Professional Quality | 25.7 |

*Note: This table provides an exploratory co-occurrence diagnostic rather than a formal validation metric. The LDA topic labels are the same K=7 topic components reported in Table 1; however, Table S1 uses soft assignment rather than dominant-topic assignment. For each review, all LDA topics with posterior probability ≥0.15 were selected and paired with every unique GPT-ABSA aspect identified in the same review. Therefore, a single review could contribute multiple topic-aspect pairs. “Selected reviews, n” indicates the number of unique reviews in which each LDA topic met the threshold. “Total topic-aspect pairs” indicates the number of review-level co-occurrences between selected LDA topics and GPT-ABSA aspects. Primary and secondary shares represent the percentage of all topic-aspect pairs for that LDA topic accounted for by the corresponding GPT-ABSA aspect. No formal cutoff is applied to classify mapping quality.*

## Table S2. Full Directed Cross-Polarity Couplings Within Cross-Aspect Mixed Reviews (All 42 Pairings)

| **Rank** | **Positive Aspect** | **Negative Aspect** | **Reviews** | **% mixed** |
| --- | --- | --- | --- | --- |
| 1 | Professional Quality | Administrative Processes | 75 | 21.5% |
| 2 | Professional Quality | Service Attitude | 70 | 20.1% |
| 3 | Professional Quality | Facility & Environment | 69 | 19.8% |
| 4 | Service Attitude | Facility & Environment | 57 | 16.3% |
| 5 | Service Attitude | Administrative Processes | 36 | 10.3% |
| 6 | Professional Quality | Inpatient Care | 25 | 7.2% |
| 7 | Inpatient Care | Facility & Environment | 21 | 6.0% |
| 8 | Professional Quality | Emergency Care | 21 | 6.0% |
| 9 | Service Attitude | Inpatient Care | 18 | 5.2% |
| 10 | Service Attitude | Professional Quality | 16 | 4.6% |
| 11 | Emergency Care | Service Attitude | 15 | 4.3% |
| 12 | Service Attitude | Emergency Care | 15 | 4.3% |
| 13 | Surgical & Specialty Care | Service Attitude | 14 | 4.0% |
| 14 | Facility & Environment | Service Attitude | 13 | 3.7% |
| 15 | Administrative Processes | Service Attitude | 12 | 3.4% |
| 16 | Facility & Environment | Administrative Processes | 12 | 3.4% |
| 17 | Surgical & Specialty Care | Professional Quality | 12 | 3.4% |
| 18 | Administrative Processes | Professional Quality | 9 | 2.6% |
| 19 | Surgical & Specialty Care | Emergency Care | 9 | 2.6% |
| 20 | Facility & Environment | Professional Quality | 8 | 2.3% |
| 21 | Professional Quality | Surgical & Specialty Care | 8 | 2.3% |
| 22 | Service Attitude | Surgical & Specialty Care | 8 | 2.3% |
| 23 | Surgical & Specialty Care | Facility & Environment | 8 | 2.3% |
| 24 | Emergency Care | Professional Quality | 7 | 2.0% |
| 25 | Surgical & Specialty Care | Inpatient Care | 7 | 2.0% |
| 26 | Administrative Processes | Facility & Environment | 6 | 1.7% |
| 27 | Facility & Environment | Inpatient Care | 6 | 1.7% |
| 28 | Emergency Care | Administrative Processes | 5 | 1.4% |
| 29 | Inpatient Care | Service Attitude | 5 | 1.4% |
| 30 | Emergency Care | Inpatient Care | 4 | 1.1% |
| 31 | Facility & Environment | Emergency Care | 4 | 1.1% |
| 32 | Inpatient Care | Administrative Processes | 4 | 1.1% |
| 33 | Inpatient Care | Emergency Care | 4 | 1.1% |
| 34 | Inpatient Care | Professional Quality | 4 | 1.1% |
| 35 | Administrative Processes | Inpatient Care | 3 | 0.9% |
| 36 | Emergency Care | Facility & Environment | 3 | 0.9% |
| 37 | Surgical & Specialty Care | Administrative Processes | 3 | 0.9% |
| 38 | Administrative Processes | Emergency Care | 2 | 0.6% |
| 39 | Facility & Environment | Surgical & Specialty Care | 2 | 0.6% |
| 40 | Administrative Processes | Surgical & Specialty Care | 1 | 0.3% |
| 41 | Emergency Care | Surgical & Specialty Care | 1 | 0.3% |
| 42 | Inpatient Care | Surgical & Specialty Care | 1 | 0.3% |

*Note: Results are computed within the cross-aspect mixed subset (n=349). Rows are ranked by review count. Reviews = number of reviews containing the specific directed positive-negative aspect pairing. % mixed = percentage of cross-aspect mixed reviews containing this pairing (Reviews / 349). Because a single review could contain multiple positively and negatively evaluated aspects, pairings are not mutually exclusive and percentages do not sum to 100%.*

## Table S3. Full Aspect-Pair Jaccard Co-occurrence Inference by Rating Group (All 21 Pairs)

| **Aspect Pair** | **J(Pos)** | **J(Neg)** | **Δ** | **95% CI** | **Perm P** | **FDR q** | **Sig.** |
| --- | --- | --- | --- | --- | --- | --- | --- |
| Inpatient Care ↔ Surgical & Specialty Care | 0.316 | 0.123 | +0.193 | [0.147, 0.241] | 0.0002 | 0.0004 | Yes |
| Professional Quality ↔ Service Attitude | 0.509 | 0.357 | +0.152 | [0.120, 0.185] | 0.0002 | 0.0004 | Yes |
| Administrative Processes ↔ Service Attitude | 0.110 | 0.255 | -0.145 | [-0.170, -0.120] | 0.0002 | 0.0004 | Yes |
| Administrative Processes ↔ Professional Quality | 0.110 | 0.231 | -0.121 | [-0.147, -0.096] | 0.0002 | 0.0004 | Yes |
| Professional Quality ↔ Surgical & Specialty Care | 0.210 | 0.089 | +0.121 | [0.095, 0.147] | 0.0002 | 0.0004 | Yes |
| Inpatient Care ↔ Professional Quality | 0.207 | 0.095 | +0.112 | [0.085, 0.138] | 0.0002 | 0.0004 | Yes |
| Emergency Care ↔ Service Attitude | 0.098 | 0.174 | -0.076 | [-0.100, -0.052] | 0.0002 | 0.0004 | Yes |
| Service Attitude ↔ Surgical & Specialty Care | 0.134 | 0.059 | +0.075 | [0.054, 0.096] | 0.0002 | 0.0004 | Yes |
| Emergency Care ↔ Professional Quality | 0.096 | 0.158 | -0.061 | [-0.086, -0.036] | 0.0002 | 0.0004 | Yes |
| Facility & Environment ↔ Professional Quality | 0.131 | 0.078 | +0.053 | [0.031, 0.075] | 0.0002 | 0.0004 | Yes |
| Inpatient Care ↔ Service Attitude | 0.156 | 0.111 | +0.045 | [0.020, 0.070] | 0.0002 | 0.0004 | Yes |
| Facility & Environment ↔ Service Attitude | 0.136 | 0.099 | +0.038 | [0.016, 0.060] | 0.0010 | 0.0018 | Yes |
| Administrative Processes ↔ Emergency Care | 0.030 | 0.067 | -0.037 | [-0.058, -0.014] | 0.0012 | 0.0019 | Yes |
| Administrative Processes ↔ Inpatient Care | 0.036 | 0.059 | -0.023 | [-0.041, -0.004] | 0.0216 | 0.0324 | Yes |
| Emergency Care ↔ Facility & Environment | 0.044 | 0.061 | -0.017 | [-0.041, 0.008] | 0.1918 | 0.2517 | No |
| Facility & Environment ↔ Surgical & Specialty Care | 0.056 | 0.040 | +0.016 | [-0.006, 0.040] | 0.1774 | 0.2483 | No |
| Administrative Processes ↔ Facility & Environment | 0.114 | 0.098 | +0.016 | [-0.015, 0.048] | 0.2566 | 0.3169 | No |
| Facility & Environment ↔ Inpatient Care | 0.102 | 0.115 | -0.013 | [-0.044, 0.020] | 0.4493 | 0.5180 | No |
| Emergency Care ↔ Surgical & Specialty Care | 0.074 | 0.063 | +0.011 | [-0.018, 0.043] | 0.4687 | 0.5180 | No |
| Emergency Care ↔ Inpatient Care | 0.109 | 0.101 | +0.009 | [-0.025, 0.041] | 0.6293 | 0.6293 | No |
| Administrative Processes ↔ Surgical & Specialty Care | 0.042 | 0.047 | -0.005 | [-0.027, 0.015] | 0.5935 | 0.6232 | No |

*Note: J(Pos) = Jaccard similarity in positive reviews (4-5 stars, n=1,961); J(Neg) = Jaccard similarity in negative reviews (1-2 stars, n=2,581); Δ = J(Pos) − J(Neg). Ranked by |Δ| in descending order. 95% CIs from 2,000 review-level bootstrap resamples. Perm P from 5,000 label permutations preserving group sizes. FDR q computed using Benjamini-Hochberg correction across all 21 pairs. Sig. = significant at FDR q < .05. Δ values are computed from unrounded Jaccard coefficients; in a few rows the displayed three-decimal inputs differ from the displayed Δ by 0.001. (Affects Emergency Care Inpatient Care, Emergency Care Professional Quality, and Facility & Environment Service Attitude.)*

## Table S4. Full Prevalence-Adjusted Co-occurrence Analysis by Rating Group

| **Aspect Pair** | **ΔJaccard** | **PMI (Positive)** | **PMI (Negative)** | **ΔPMI** |
| --- | --- | --- | --- | --- |
| Inpatient Care ↔ Surgical & Specialty Care | +0.193 | +1.15 | +1.25 | -0.10 |
| Professional Quality ↔ Service Attitude | +0.152 | +0.03 | +0.11 | -0.07 |
| Administrative Processes ↔ Service Attitude | -0.145 | -0.10 | -0.27 | +0.17 |
| Administrative Processes ↔ Professional Quality | -0.121 | -0.13 | -0.12 | -0.01 |
| Professional Quality ↔ Surgical & Specialty Care | +0.121 | +0.21 | +0.40 | -0.19 |
| Inpatient Care ↔ Professional Quality | +0.112 | -0.10 | -0.14 | +0.04 |
| Emergency Care ↔ Service Attitude | -0.076 | +0.08 | +0.20 | -0.12 |
| Service Attitude ↔ Surgical & Specialty Care | +0.075 | -0.30 | -0.22 | -0.08 |
| Emergency Care ↔ Professional Quality | -0.061 | +0.03 | +0.23 | -0.20 |
| Facility & Environment ↔ Professional Quality | +0.053 | -0.38 | -0.73 | +0.34 |
| Inpatient Care ↔ Service Attitude | +0.045 | -0.40 | -0.05 | -0.34 |
| Facility & Environment ↔ Service Attitude | +0.038 | -0.29 | -0.56 | +0.27 |
| Administrative Processes ↔ Emergency Care | -0.037 | -0.91 | -0.89 | -0.03 |
| Administrative Processes ↔ Inpatient Care | -0.023 | -1.26 | -0.78 | -0.49 |
| Emergency Care ↔ Facility & Environment | -0.017 | -0.62 | -0.54 | -0.08 |
| Facility & Environment ↔ Surgical & Specialty Care | +0.016 | -0.84 | -0.40 | -0.43 |
| Administrative Processes ↔ Facility & Environment | +0.016 | +0.42 | -0.42 | +0.84 |
| Facility & Environment ↔ Inpatient Care | -0.013 | -0.23 | +0.52 | -0.74 |
| Emergency Care ↔ Surgical & Specialty Care | +0.011 | +0.09 | +0.23 | -0.14 |
| Emergency Care ↔ Inpatient Care | +0.009 | +0.48 | +0.37 | +0.12 |
| Administrative Processes ↔ Surgical & Specialty Care | -0.005 | -0.93 | -0.46 | -0.47 |

*Note: Positive reviews are rated 4-5 stars (n=1,961), and negative reviews are rated 1-2 stars (n=2,581). PMI = log2(P(A∩B) / (P(A) x P(B))) computed within each rating group. ΔPMI = PMI(Positive) - PMI(Negative). Rows are sorted by absolute ΔJaccard to match the main-text sensitivity analysis.*
